# Biomechanical Phenotyping of Forced Expiration for Precision Pulmonary Rehabilitation: A Machine Learning Approach to Identify Structural and Kinetic Drivers

**DOI:** 10.3390/arm94020026

**Published:** 2026-04-17

**Authors:** Noppharath Sangkarit, Weerasak Tapanya

**Affiliations:** Department of Physical Therapy, School of Allied Health Sciences, University of Phayao, Phayao 56000, Thailand; noppharath.sa@up.ac.th

**Keywords:** spirometry, biomechanics, machine learning, phenotyping, respiratory diagnostics

## Abstract

**Highlights:**

**What are the main findings?**

- Machine learning analysis of standard spirometry data revealed three biomechanical phenotypes of forced expiration: Load-Constrained, Mechanically Efficient, and Dynamic Collapse.
- A neural network model demonstrated that structural mass constraints and dynamic airway instability predict clinical respiratory impairments (93.2% accuracy) significantly better than conventional demographics such as chronological age or biological sex.

**What are the implications of the main findings?**

- Translating routine volumetric spirometry into kinetic and structural indices shifts the diagnostic paradigm from merely identifying volume loss to pinpointing the specific underlying mechanical failures.
- Classifying patients by these functional phenotypes facilitates precision cardiopulmonary rehabilitation, enabling clinicians to prescribe highly targeted interventions—such as airway patency strategies or thoracic mobility exercises—rather than generic aerobic conditioning.

**Abstract:**

Background: Standard spirometry fundamentally overlooks the mechanical dynamics of forced expiration. This study derived novel biomechanical parameters to establish functional phenotypes and predict clinical respiratory impairments. Methods: Utilizing 16,596 acceptable spirometry records from NHANES (2007 to 2012), parameters reflecting kinetic power, mass constraint, and airway instability were mathematically derived. Principal component analysis, K-means clustering, and a Multilayer Perceptron neural network were sequentially applied. Results: Three distinct biomechanical phenotypes emerged: Load-Constrained (45.4%), Mechanically Efficient (23.5%), and Dynamic Collapse (31.0%). Aging significantly degraded kinetic power, demonstrating a steeper functional decline in males (*p* < 0.001). The neural network achieved 93.2% testing accuracy in classifying spirometric abnormalities. Crucially, Dynamic Airway Collapse Ratio (100% normalized importance), BMI (89.4%), and kinetic power (86.2%) fundamentally outperformed traditional demographic predictors such as chronological age (20.4%) and biological sex (7.1%). Conclusions: Structural and dynamic kinetic factors drive pulmonary dysfunction far more accurately than conventional demographics. Classifying these mechanical phenotypes facilitates highly targeted precision cardiopulmonary rehabilitation.

## 1. Introduction

Forced expiration is universally recognized as a fundamental clinical metric for evaluating pulmonary function. This physiological action is typically assessed through spirometry to capture total exhaled volumes and peak airflow rates [1,2]. While traditional spirometric metrics, such as forced expiratory volume in one second and forced vital capacity, are indispensable for diagnosing obstructive and restrictive pulmonary diseases [3], they primarily reflect macroscopic volumetric outcomes. From the perspective of human movement science, forced expiration is not merely a passive deflation of the lungs but a highly coordinated biomechanical motor task [4]. It demands explosive kinetic power from the core and abdominal musculature, seamlessly synchronized with the structural compliance and elastic recoil of the thoracic cage [4,5,6].

Despite the complex neuromuscular coordination required for this task, current interpretive strategies in pulmonary diagnostics rely almost entirely on statistical reference equations adjusted for chronological age, biological sex, and standing height [7,8]. This conventional approach fundamentally overlooks the intricate mechanical interplay between muscular force generation and anatomical constraints. For instance, generalized body mass acts as a physical impedance that the expiratory muscles must actively overcome to expand and forcefully contract the thorax [9,10]. Furthermore, the dynamic stability of the airways during a forceful exhalation is strictly dictated by the structural integrity of the tracheobronchial tree against massive intrathoracic pressures [11]. Relying exclusively on scalar volume outputs fails to capture these complex dynamic and structural constraints, thereby masking the true biomechanical mechanisms responsible for respiratory impairments.

Despite extensive advancements in respiratory physiology and human movement science, the application of advanced biomechanical modeling to standard clinical spirometric data remains largely unexplored. Previous biomechanical studies have successfully utilized principal component analysis to decompose complex human locomotion and postural control into fundamental movement synergies [12,13]. Yet, similar dimensionality reduction techniques have not been systematically applied to respiratory mechanics to identify functional movement phenotypes. Furthermore, it is clinically evident that respiratory decline is not purely a function of chronological aging [14]. Instead, the functional capacity of the respiratory system is highly influenced by mechanical loads, such as increased body mass index, and inherent anatomical dimorphisms between sexes [15,16]. Therefore, a critical knowledge gap exists regarding how thoracic mass constraints and kinetic power interact to drive age related respiratory decline. It remains unknown whether these specific biomechanical factors can predict clinical impairments more accurately than traditional demographic variables.

To bridge this substantial research gap, standard spirometric data and basic anthropometric measures can be mathematically transformed into novel biomechanical parameters. By conceptualizing peak expiratory flow as peak kinetic velocity and exhaled volume as mechanical displacement, the kinetic power proxy of the expiratory muscles can be quantified. Similarly, the ratio of body mass to vital capacity can serve as an objective index of thoracic mechanical impedance. Translating clinical respiratory data into these structural and kinetic metrics provides a profound biomechanical rationale for understanding pulmonary dysfunction. This innovative approach shifts the diagnostic paradigm from merely identifying a mathematical loss of volume to pinpointing the specific mechanical failure. This failure could manifest as a lack of neuromuscular motor power, an excessive thoracic mass constraint, or severe dynamic airway collapse [17,18].

The primary objective of the present study was to derive novel biomechanical respiratory parameters from a large population based cohort and utilize unsupervised machine learning to classify distinct functional respiratory phenotypes. A secondary objective was to evaluate the interactive effects of biological sex and chronological aging on these biomechanical traits. A final objective was to determine the predictive superiority of these mechanical parameters over conventional demographics using artificial neural networks. It was hypothesized that (1) complex respiratory mechanics could be mathematically decomposed into identifiable biomechanical synergies and distinct clinical phenotypes, (2) the trajectory of age related biomechanical decline would progress at significantly different rates between males and females independent of baseline body mass, and (3) structural and dynamic biomechanical parameters would fundamentally outperform chronological age and sex in predicting clinical spirometric abnormalities.

The clinical application of these proposed biomechanical phenotypes holds substantial promise for precision rehabilitation. By classifying patients based on their specific mechanical deficits rather than generalized volumes, clinicians and physical therapists can design highly targeted cardiopulmonary rehabilitation programs [19]. For example, identifying a patient with a load constrained phenotype would prioritize weight management and thoracic mobility exercises. Conversely, a dynamic collapse phenotype would necessitate interventions focused on specific breathing control techniques and airway clearance maneuvers. Ultimately, uncovering these biomechanical traits will facilitate targeted precision medicine in respiratory care and human movement rehabilitation.

## 2. Materials and Methods

### 2.1. Study Design, Data Source, and Ethical Considerations

A retrospective, cross-sectional analysis was conducted using a publicly accessible, fully de-identified dataset from the National Health and Nutrition Examination Survey (NHANES) 2007–2012 cohorts [20,21,22]. Because this study involved the secondary analysis of anonymized public data, Institutional Review Board (IRB) approval was exempt. However, the original NHANES data collection protocols were formally approved by the National Center for Health Statistics (NCHS) Research Ethics Review Board, with written informed consent obtained from all participants prior to enrollment [20]. The dataset included five self-identified racial categories (White, Black, Mexican American, Other Hispanic, and Mixed), which were analyzed collectively for methodological simplicity.

To maintain rigorous analytical standards and ensure robust biomechanical modeling, the current investigation included only participants (aged 6 to 80 years) who provided high-quality spirometry data. Specifically, inclusion was restricted to individuals achieving American Thoracic Society/European Respiratory Society (ATS/ERS) Grade “A” and “B” quality ratings. This criterion ensured that each participant had a minimum of three acceptable spirometric maneuvers and two reproducible curves with observed values within a 150 mL variance [20,21]. Extreme outliers resulting from technical artifacts were systematically removed.

The curated dataset yielded 16,596 valid records for advanced analyses. To enhance clinical relevance and establish a baseline for comparison, the cohort was subsequently categorized into conventional diagnostic patterns (normal, obstructive, restrictive, and mixed) based on standard spirometry and the Lower Limit of Normal (LLN) criteria. The study population exhibited a perfectly balanced sex distribution, comprising 8293 males (50.0%) and 8303 females (50.0%). Chronologically, the cohort spanned a wide age range, with the largest proportion falling within the 10–19 years age group (21.9%), followed by relatively uniform distributions across the subsequent adult decades: 20–29 years (13.2%), 30–39 years (13.4%), 40–49 years (13.0%), 50–59 years (11.9%), and 60–69 years (10.9%). Children aged 6–9 years and older adults aged 70–79 years represented 9.2% and 6.4% of the sample, respectively. Baseline anthropometric assessments reflected a highly diverse population, with body mass ranging from 16.4 to 218.2 kg, standing height from 104.6 to 203.8 cm, and body mass index (BMI) spanning 12.5 to 84.9 kg/m^2^.

### 2.2. Standard Spirometric Data Acquisition

Spirometric assessments across the NHANES 2007–2012 cohorts were uniformly conducted utilizing a dry rolling cylinder spirometer (Ohio Medical Instrument Company, Cincinnati, OH, USA), adhering strictly to the 2005 ATS/ERS technical standards [20,21,23]. Baseline raw variables extracted for the formulation of biomechanical models included Peak Expiratory Flow (PEF, L/s), Forced Expiratory Volume in 1 s (FEV_1_, L), Forced Vital Capacity (FVC, L), Forced Expiratory Flow at 25–75% of FVC (FEF_25–75_, L/s), and Forced Expiratory Time (FET, s).

### 2.3. Derivation of Novel Biomechanical Respiratory Parameters

A major limitation of traditional spirometric interpretation is its reliance on isolated volume and flow metrics, which often fail to fully encapsulate the dynamic mechanical constraints of the thorax and the kinetic efficiency of core expiratory muscles. To address this methodological gap, standard spirometric data and anthropometric measures were mathematically transformed into novel biomechanical parameters conceptualized to reflect mechanical power, structural mass impedance, and dynamic airway stability.

First, the Biomechanical Expiratory Power Proxy (BEPP, L^2^/s·m) was computed to represent the mechanical kinetic power generated by the expiratory muscles. This variable integrates peak velocity and displacement volume, normalized by body height to account for anatomical scaling, and is calculated as:

$$BEPP=\frac{PEF\times {FEV}_{1}}{Height}$$

To evaluate the mechanical impedance or load exerted by body mass against thoracic expansion, the Thoracic Mass-to-Volume Constraint (TMVC, kg/m^2^·L) was formulated. A higher TMVC indicates a greater mechanical burden required to mobilize one liter of lung volume, derived using the equation:

$$TMVC=\frac{BMI}{FVC}$$

Furthermore, the Dynamic Airway Collapse Ratio (DACR, dimensionless) was established as an indicator of structural airway instability. This ratio compares the initial explosive flow to the mid-expiratory flow, where an elevated value suggests rapid mid-expiratory narrowing, calculated as:

$$DACR=\frac{PEF}{{FEV}_{25-75}}$$

The kinetic loss of flow velocity after peak exertion was quantified by the Mid-Expiratory Flow Deceleration (MEFD, L/s), where higher deceleration values signify greater resistance in the distal airways, expressed as:

$$MEFD=PEF-{FEV}_{25-75}$$

Finally, to reflect neuromuscular motor control efficiency, the Expiratory Drive Efficiency (EDE, L/s) was defined as the volume of air successfully expelled per unit of total forced expiratory time, calculated as:

$$EDE=\frac{{FEV}_{1}}{FET}$$

### 2.4. Statistical Analysis and Machine Learning Protocol

All data processing, multivariate analyses, and predictive modeling were executed using IBM SPSS Statistics software (version 30.0 for Mac, SPSS Inc., Chicago, IL, USA). The analytical framework was conducted in sequential phases.

#### 2.4.1. Principal Component Analysis (PCA)

To uncover underlying respiratory movement synergies, a PCA with Varimax rotation and Kaiser Normalization was applied to the five derived biomechanical variables. The extraction criterion was based on eigenvalues greater than 1.0. The resultant principal components (PCs) effectively reduced dimensionality and extracted orthogonal factors representing “Expiratory Power and Thoracic Compliance” and “Dynamic Airway Instability.” Standardized regression factor scores were preserved for subsequent classification.

#### 2.4.2. Biomechanical Phenotyping via K-Means Clustering

To translate the continuous PC scores into clinically meaningful profiles, a K-means cluster analysis was performed. An initial algorithm utilizing k = 4 was employed to identify and exclude extreme technical outliers (n = 1). A final predetermined cluster configuration of k = 3 was subsequently executed to classify the population into distinct functional phenotypes based on their mechanical efficiency and structural constraints.

#### 2.4.3. Multivariate Analysis of Covariance (MANCOVA)

To investigate the independent and interactive effects of biological sex and chronological age (Age_Groups) on respiratory biomechanics, a one-way MANCOVA was conducted. BMI was incorporated as a covariate to isolate the effects of aging and sex from generalized body mass variations. Pillai’s trace was reported due to its robustness in large sample sizes. Post hoc pairwise comparisons were adjusted using the Bonferroni correction, and effect sizes were determined utilizing partial eta-squared ($\eta_{p}^{2}$).

#### 2.4.4. Multilayer Perceptron (MLP) Neural Network

To determine the hierarchical predictive superiority of the newly derived biomechanical parameters against conventional demographic characteristics (Age, Sex, Race), an MLP artificial neural network was constructed. The clinical target variable was the presence of spirometric abnormalities based on segmented prediction models established in the foundational dataset [20]. The dataset was randomly partitioned into a training set (70%) and an independent testing set (30%) to prevent overfitting. The network architecture was optimized using a hyperbolic tangent activation function in the hidden layer and a Softmax activation function in the output layer. Normalized independent variable importance was computed to rank the predictive power of each parameter. A significance level of α = 0.05 was maintained for all inferential statistics.

## 3. Results

### 3.1. Principal Respiratory Synergies

To identify underlying biomechanical patterns during forced expiration, a principal component analysis (PCA) with Varimax rotation was performed on the novel kinetic and structural variables. The analysis extracted two orthogonal principal components (PCs) that collectively accounted for 69.63% of the total variance in respiratory mechanics. As detailed in Table 1, the first component (PC1) explained 46.36% of the variance and was designated as “Expiratory Power and Thoracic Compliance”. This synergy was characterized by strong positive loadings for the Biomechanical Expiratory Power Proxy (BEPP) and Mid-Expiratory Flow Deceleration (MEFD), coupled with a strong negative loading for the Thoracic Mass-to-Volume Constraint (TMVC). The second component (PC2) explained 23.26% of the variance and was termed “Dynamic Airway Instability”, driven primarily by a high positive loading for the Dynamic Airway Collapse Ratio (DACR) and a negative loading for Expiratory Drive Efficiency (EDE).

### 3.2. Biomechanical Phenotypes of Forced Expiration

**Figure 1:**
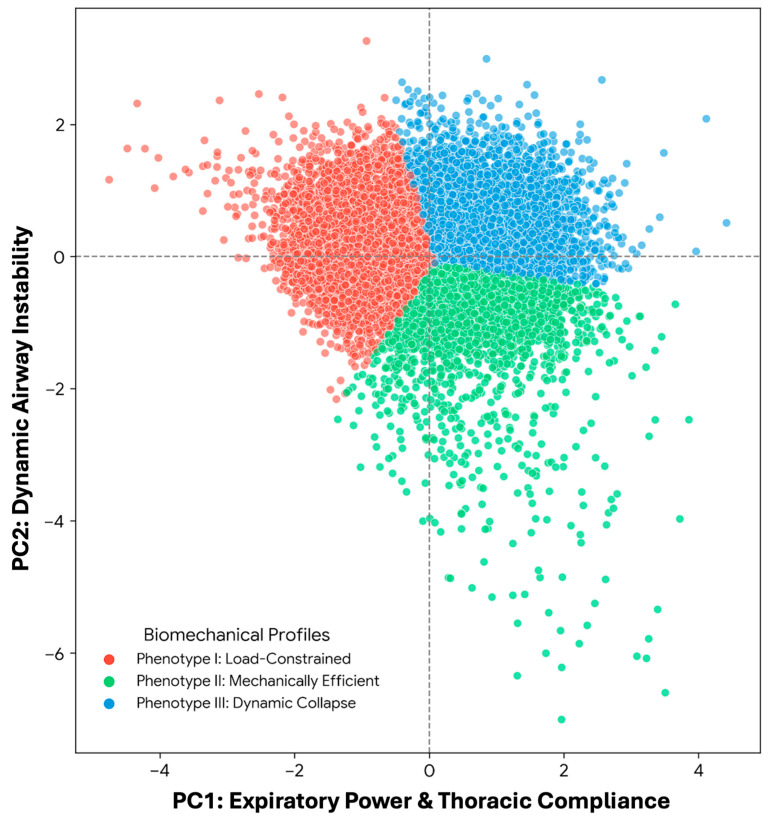
Biomechanical phenotypes of forced expiration derived from principal component analysis. The scatter plot illustrates the distribution of 16,595 individuals across two primary biomechanical dimensions: PC1 (Expiratory Power & Thoracic Compliance) and PC2 (Dynamic Airway Instability). K-means clustering identified three distinct functional profiles: Phenotype I (Load-Constrained; red) characterized by high mass constraint and low power; Phenotype II (Mechanically Efficient; green) demonstrating optimal kinetic drive and structural stability; and Phenotype III (Dynamic Collapse; blue) exhibiting adequate initial power but marked mid-expiratory airway narrowing.

Following the extraction of movement synergies, a K-means cluster analysis was utilized to categorize the continuous respiratory mechanics (PC1 and PC2 scores) into distinct functional phenotypes. Out of 16,596 valid cases, one extreme outlier was excluded to maintain model robustness. The algorithm successfully identified three primary biomechanical profiles (Table 2 and Figure 1). The largest group, Phenotype I (*n* = 7534), demonstrated diminished expiratory power and high thoracic constraints, representing a “Load-Constrained” profile. Phenotype II (*n* = 3909) exhibited above-average kinetic power and optimal airway stability, classified as the “Mechanically Efficient” profile. Conversely, Phenotype III (*n* = 5152) showed adequate initial drive but marked mid-expiratory airway narrowing, indicating a “Dynamic Collapse” profile.

When applying the conventional LLN criteria to the overall cohort, the subjects were distributed into normal (n = 11,452), obstructive (n = 2157), restrictive (n = 1988), and mixed (n = 999) patterns. Documenting this baseline clinical distribution allows for a direct comparison between established diagnostic categories and the newly identified biomechanical phenotypes.

### 3.3. Effects of Sex and Aging on Respiratory Biomechanics

**Figure 2:**
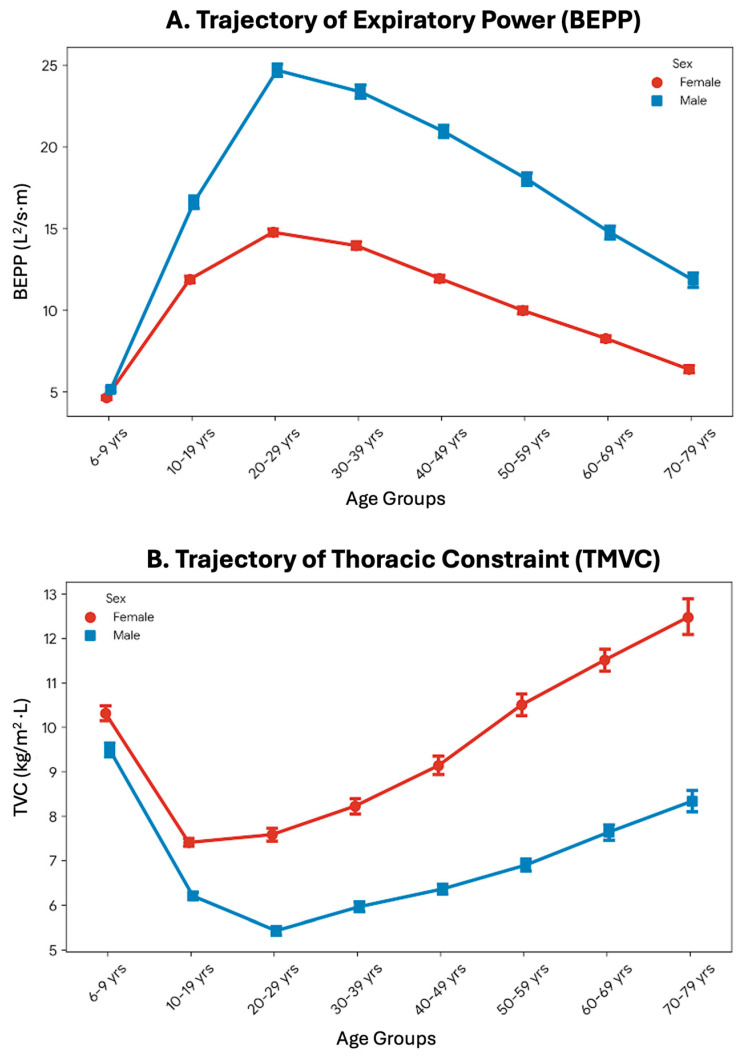
Interactive effects of biological sex and chronological age on respiratory biomechanics. Line graphs present the estimated marginal means (with 95% confidence intervals) for (**A**) Biomechanical Expiratory Power Proxy (BEPP) and (**B**) Thoracic Mass-to-Volume Constraint (TMVC) across different age groups, separated by sex. The trajectories demonstrate a significant Sex × Age_Groups interaction (*p* < 0.001). While both sexes experience an age-related decline in expiratory power and an increase in thoracic constraint, the rates of these biomechanical alterations differ significantly between males (blue circles) and females (red squares), independent of baseline body mass index.

A one-way multivariate analysis of covariance (MANCOVA) was conducted to evaluate the independent and interactive effects of sex and chronological age on the biomechanical parameters, while statistically controlling for body mass index (BMI). The multivariate results indicated significant main effects for both Age_Groups (Pillai’s trace = 0.889, *p* < 0.001, partial η^2^ = 0.178) and Sex on the combined variables. Univariate analyses (Table 3) revealed that the aging process exerted a profound effect on thoracic constraint (TMVC; partial η^2^ = 0.429) and kinetic power (BEPP; partial η^2^ = 0.369). Biological sex similarly demonstrated substantial main effects on BEPP and TMVC. Crucially, a significant Sex × Age_Groups interaction was observed across all parameters, indicating that the trajectory of age-related biomechanical decline progresses at different rates between males and females, independent of baseline body mass (Figure 2).

### 3.4. Neural Network Prediction of Respiratory Impairments

**Figure 3:**
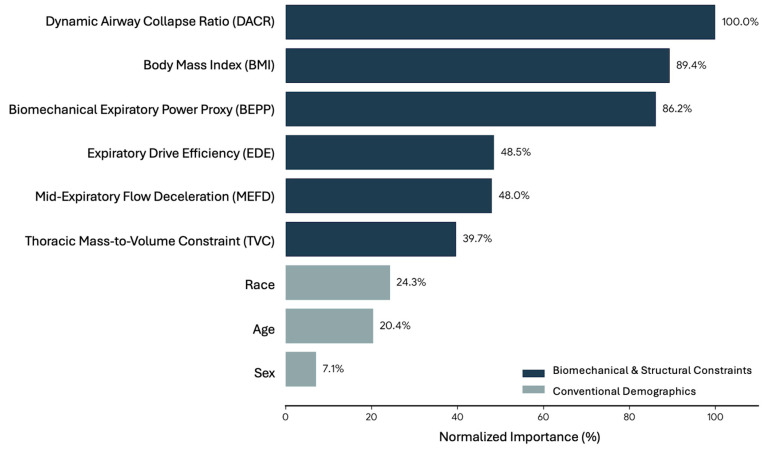
Normalized predictor importance from the Multilayer Perceptron (MLP) neural network. The horizontal bar chart displays the relative contribution of each independent variable in predicting clinical spirometric abnormalities. Biomechanical and structural load factors (navy blue bars), notably the Dynamic Airway Collapse Ratio (DACR, 100%), BMI (89.4%), and BEPP (86.2%), demonstrated substantially higher predictive power compared to conventional demographic factors (gray bars) such as race, age, and sex.

To determine the predictive superiority of the novel biomechanical parameters against conventional demographic factors, a Multilayer Perceptron (MLP) artificial neural network was trained to classify clinical spirometric abnormalities. The dataset was partitioned into a training sample (70.2%, *n* = 11,648) and an independent testing sample (29.8%, *n* = 4948). The MLP model achieved a high overall classification accuracy of 93.55% in the training phase and successfully generalized to the testing dataset with 93.16% accuracy. The independent variable importance analysis (Table 4 and Figure 3) revealed that dynamic and structural biomechanical factors fundamentally outperformed traditional demographic traits. Dynamic Airway Collapse Ratio (DACR) and BMI emerged as the most critical predictors, whereas chronological age, race, and sex provided substantially less predictive weight.

## 4. Discussion

The clinical interpretation of pulmonary function has traditionally relied on volumetric metrics and generic demographic reference equations. However, this conventional approach fundamentally overlooks the complex neuromuscular and structural mechanics required to perform a forced expiration. The present study aimed to reconceptualize standard spirometry through the lens of human movement science by deriving novel biomechanical parameters. The analyses successfully decomposed forced expiration into two primary mechanical synergies, leading to the identification of three distinct biomechanical phenotypes. Furthermore, the findings demonstrated that age related mechanical decline is highly dependent on biological sex. Crucially, artificial neural network modeling revealed that dynamic and structural biomechanical factors drastically outperformed chronological age and biological sex in predicting clinical spirometric abnormalities.

The identification of three functional respiratory phenotypes provides a profound mechanical explanation for pulmonary impairments. Phenotype I, designated as the Load Constrained profile, represents a distinct mechanical disadvantage where thoracic mass heavily impedes lung expansion and contraction. From a biomechanical perspective, increased body mass surrounding the rib cage directly alters the compliance of the chest wall [24]. This external mass acts as a continuous resistive load. Consequently, the core and abdominal musculature must generate substantially higher kinetic energy simply to overcome gravity and tissue inertia [25,26,27]. When the kinetic output, represented by the Biomechanical Expiratory Power Proxy (BEPP), is insufficient to overcome the Thoracic Mass to Volume Constraint (TMVC), expiratory flow is fundamentally compromised, leading to a restrictive like physiological state. Conversely, Phenotype III, classified as the Dynamic Collapse profile, illustrates a failure in structural airway integrity rather than a lack of muscular power. In human movement science, explosive motor tasks require absolute structural stability to transfer kinetic energy efficiently. During forced expiration, high initial kinetic power generates massive positive intrapleural pressure. According to fluid dynamics and the equal pressure point theory, if the structural integrity of the distal airways cannot withstand this massive transmural pressure, the bronchioles undergo premature dynamic compression [28,29,30]. This mechanical failure creates a sharp deceleration in mid expiratory flow, which was perfectly captured by the Dynamic Airway Collapse Ratio (DACR) in the present study. This finding highlights that strong core muscles alone are insufficient if the underlying structural tubes lack the rigidity to remain patent under pressure. Specifically for this phenotype, the observed dynamic collapse and mid-expiratory flow deceleration (captured by DACR and MEFD) are fundamentally influenced by the dynamic micro-mechanical interaction between airway smooth muscle (ASM) cells and the extracellular matrix (ECM). Alterations in the structural integrity of collagen, elastin, and proteoglycans within the ECM can severely compromise cellular-level rigidity, thereby exacerbating airway narrowing during high-pressure maneuvers and driving the biomechanical failure seen in this profile.

The trajectory of biomechanical decline observed in the multivariate analysis revealed a significant interaction between biological sex and chronological age. While both sexes experienced an inevitable increase in thoracic constraint and a simultaneous decrease in expiratory power over time, the rate of deterioration differed significantly. Biomechanically, the aging process is closely associated with sarcopenia, which particularly affects the fast twitch muscle fibers of the abdominal wall that are critical for explosive expiratory maneuvers [31,32,33]. The steeper decline in kinetic power observed in males may be attributed to a higher baseline muscle mass that undergoes a more pronounced absolute reduction during the aging process [34]. Furthermore, age related stiffening of the costovertebral joints and calcification of costal cartilages alter rib cage kinematics, compounding the mechanical constraint and reducing overall respiratory efficiency in older adults [35,36].

The predictive superiority of mechanical variables over demographic factors in the Multilayer Perceptron model represents a crucial paradigm shift in respiratory diagnostics. Chronological age and biological sex are merely surrogate markers of physiological decline [37,38]. They do not directly cause respiratory impairment. Instead, the actual root causes of functional limitation are the physical alterations in tissue compliance, airway resistance, and neuromuscular power [39]. By mathematically extracting these underlying mechanical forces, the neural network could accurately classify pathological states with high precision. This confirms that physical mechanics dictate pulmonary function far more accurately than the mere passage of time or genetic sex.

These findings hold substantial clinical value for precision rehabilitation. By identifying a patient based on their specific biomechanical phenotype, physical therapists and clinicians can prescribe highly targeted interventions [40]. Patients exhibiting a Load Constrained profile would benefit most from weight management, core muscle strengthening, and specific thoracic mobility exercises to reduce mechanical impedance. In contrast, patients categorized into the Dynamic Collapse phenotype require strategies designed to maintain airway patency and regulate intra thoracic pressure, such as pursed lip breathing or positive expiratory pressure therapy, rather than generic aerobic conditioning [41].

While our BEPP and TMVC ratios provide clear mechanical indices, it is crucial to acknowledge that they represent a linear simplification of a highly complex system. Seminal studies [42,43,44,45] have demonstrated that the respiratory system is fundamentally governed by non-linear viscoelastic properties and active load compensation mechanisms. During a forced expiration, the relationship between muscular force and flow velocity is inherently distorted by ‘active elastance’ and post-inspiratory braking of the diaphragm. Consequently, these physiological adaptations can render simple biomechanical ratios non-linear, depending heavily on the dynamic state and continuous deformation of the thorax. Furthermore, it is important to contextualize these spirometry-derived parameters with the concept of mechanical impedance measured via the forced oscillation technique (FOT). While our proposed model identifies macroscopic kinetic drivers, FOT provides a direct measurement of the complex, frequency-dependent impedance that characterizes various lung pathologies. Integrating this concept further explains the inherent non-linear relationships between expiratory flow, volume, and mass constraints that standard spirometry alone cannot fully capture.

Several limitations of the present study must be acknowledged. The retrospective design utilizing secondary survey data prevented the direct measurement of muscle activation via electromyography or intra thoracic pressure via esophageal balloons. Additionally, the cross-sectional nature of the dataset limits the ability to establish true longitudinal causality regarding age related mechanical decline. Furthermore, the mathematical derivation of biomechanical parameters from volumetric data, while highly predictive, serves as a proxy rather than a direct kinetic measurement. Future research should prospectively validate these newly derived biomechanical parameters using advanced kinematic motion capture and direct kinetic assessments. Furthermore, longitudinal tracking of these biomechanical phenotypes across specific clinical populations (e.g., COPD or restrictive lung disease cohorts) is essential to validate their prognostic value and sensitivity to therapeutic interventions over time.

## 5. Conclusions

In conclusion, the present study successfully reconceptualized standard spirometry into an advanced biomechanical framework. By deriving novel metrics of kinetic power and structural constraint, three distinct functional respiratory phenotypes were established. The evidence clearly demonstrates that physical mass constraints and dynamic airway instability are the true primary drivers of respiratory impairment, fundamentally outperforming traditional demographic predictors such as age and sex. This innovative biomechanical perspective shifts the diagnostic focus from simple volume loss to the specific mechanical failures underlying the condition. Ultimately, this approach provides a robust, mechanistic foundation that paves the way for highly individualized and precision targeted cardiopulmonary rehabilitation strategies.

## Figures and Tables

**Table 1:** Principal component loadings for the novel biomechanical respiratory variables.

Biomechanical Variable	PC1: Expiratory Power and Thoracic Compliance	PC2: Dynamic Airway Instability
Biomechanical Expiratory Power Proxy (BEPP)	**0.931**	−0.111
Mid-Expiratory Flow Deceleration (MEFD)	**0.791**	0.402
Thoracic Mass-to-Volume Constraint (TMVC)	**−0.751**	0.230
Dynamic Airway Collapse Ratio (DACR)	0.093	**0.735**
Expiratory Drive Efficiency (EDE)	0.468	**−0.657**

Note: Extraction Method: Principal Component Analysis. Rotation Method: Varimax with Kaiser Normalization. Loadings with an absolute value > 0.60 are highlighted in bold.

**Table 2:** Final cluster centers and distribution of the identified biomechanical phenotypes.

Phenotype Classification	n (%)	PC1 Center (Power & Compliance)	PC2 Center (Airway Instability)
**Phenotype I:** Load-Constrained	7534 (45.4%)	−0.858	0.075
**Phenotype II:** Mechanically Efficient	3909 (23.5%)	0.587	−0.880
**Phenotype III:** Dynamic Collapse	5152 (31.0%)	0.806	0.543

**Table 3:** MANCOVA results detailing the main and interactive effects on biomechanical variables, adjusting for BMI.

Source	Dependent Variable	df	F-Value	*p*-Value	Partial η^2^
Age_Groups	TMVC	7	1779.32	<0.001	0.429
	BEPP	7	1384.49	<0.001	0.369
	MEFD	7	1199.87	<0.001	0.336
Sex	TMVC	1	6245.19	<0.001	0.274
	BEPP	1	6191.55	<0.001	0.272
Sex × Age_Groups	BEPP	7	170.71	<0.001	0.067
	TMVC	7	160.23	<0.001	0.063

**Table 4:** Independent variable importance from the Multilayer Perceptron (MLP) model.

Predictor Variable	Importance	Normalized Importance (%)
Dynamic Airway Collapse Ratio (DACR)	0.216	100.0%
Body Mass Index (BMI)	0.193	89.4%
Biomechanical Expiratory Power Proxy (BEPP)	0.186	86.2%
Expiratory Drive Efficiency (EDE)	0.105	48.5%
Mid-Expiratory Flow Deceleration (MEFD)	0.104	48.0%
Thoracic Mass-to-Volume Constraint (TMVC)	0.086	39.7%
Race	0.052	24.3%
Age	0.044	20.4%
Sex	0.015	7.1%

## Data Availability

The datasets analyzed during the current study are freely accessible as an open-access resource. The refined secondary dataset, originally curated from the NHANES 2007–2012 operational cycles by Zavorsky (2024) [20], is hosted as an open-access resource in the Mendeley Data repository (https://doi.org/10.17632/dwjykg3xww.1). This repository contains the comprehensive demographic profiles, baseline anthropometric dimensions, and standardized spirometric records necessary for replicating the biomechanical phenotyping and predictive modeling presented in this investigation.
